# Impact of an intergenerational program to improve loneliness and social isolation in older adults initiated at the time of emergency department discharge: study protocol for a three-arm randomized clinical trial

**DOI:** 10.1186/s13063-024-08250-2

**Published:** 2024-06-28

**Authors:** David Zheng, Louise Rose, Bjug Borgundvaag, Shelley L. McLeod, Donald Melady, Rohit Mohindra, Samir Sinha, Virginia Wesson, Lesley Wiesenfeld, Sabrina Kolker, Alex Kiss, Judy A. Lowthian, Jacques S. Lee

**Affiliations:** 1 Schulich School of Medicine and Dentistry, 1151 Richmond St, London, ON N6A 5C1 Canada; 2 Faculty of Nursing, Midwifery and Palliative Care, King’s College London, London, UK; 3 Schwartz/Reisman Emergency Medicine Institute, Sinai Health, Toronto, ON Canada; 4 Mount Sinai Hospital, Sinai Health, Toronto, ON Canada; 5 University of Toronto, Toronto, ON Canada; 6 Department of Emergency Medicine, North York General Hospital, Toronto, ON Canada; 7 Granovsky-Gluskin Family Medicine Centre, Mount Sinai Hospital, Sinai Health, Toronto, ON Canada; 8 Department of Epidemiology and Biostatistics, Sunnybrook Research Institute, Toronto, ON Canada; 9 Bolton Clarke Research Institute, Melbourne, VIC Australia; 10 School of Public Health and Preventive Medicine, Monash University, Melbourne, VIC Australia

**Keywords:** Social isolation, Loneliness, Randomized controlled trial, Emergency department, Intergenerational, Geriatrics, Aging, Volunteering

## Abstract

**Background:**

Social isolation and loneliness (SIL) worsens mortality and other outcomes among older adults as much as smoking. We previously tested the impact of the HOW R U? intervention using peer support from similar-aged volunteers and demonstrated reduced SIL among older adults discharged from the emergency department (ED). Generativity, defined as “the interest in establishing and guiding the next generation,” can provide an alternative theoretical basis for reducing SIL via intergenerational programs between members of younger and older generations. The current protocol will examine the impact of younger intergenerational volunteers providing the HOW RU? intervention.

**Methods:**

In this randomized clinical trial, we will compare the following three arms: (1) the standard same-generation peer support HOW R U? intervention, (2) HOW R U? intervention delivered by intergenerational volunteers, and (3) a common wait-list control group. Outcome assessors will be blinded to the intervention. Trained volunteers will deliver 12 weekly telephone support calls. We will recruit participants ≥ 70 years of age with baseline loneliness (six-item De Jong loneliness score of 2 or greater) from two EDs. Research staff will assess SIL, depression, quality of life, functional status, generativity, and perceived benefit at baseline, at 12 weeks, and 24 weeks post-intervention.

**Discussion:**

We hypothesize participants receiving the intergenerational intervention will show improved outcomes compared to the control group and peer support HOW R U? intervention. We also hypothesize that participants with higher perceptions of generativity will have greater reductions in SIL than their lower generativity counterparts. Aging is experienced diversely, and social interventions combatting associated SIL should reflect that diversity. As part of a program of research following the Obesity-Related Behavioral Intervention Trials (ORBIT) model, the findings of this RCT will be used to define which intervention characteristics are most effective in reducing SIL.

**Trial registration:**

ClinicalTrials.gov NCT05998343 Protocol ID:21-0074E. Registered on 24 July 2023.

## Background

Older adults use emergency services at higher rates and also are more likely to experience social isolation and loneliness (SIL) than younger people [1]. Socially isolated and lonely older adults have an increased risk of all-cause mortality rivaling that of risk factors such as obesity and smoking, as well as an increased rate of admission to long-term care and unplanned emergency hospitalization [2–4]. Facing an aging population, preventative measures to address this important public health issue are needed, and the emergency department (ED) is a novel setting to identify SIL and to intervene [5, 6]. Lowthian et al. conducted a pilot trial of a support program called “HOW R U?” for older adults after discharge from the ED which showed reduced symptoms of depression and SIL from baseline among older adults [4]. Our research group is currently conducting a three-arm RCT comparing the same-generation peer support “HOW R U?” intervention delivered by telephone or video call to a common control group (clinicaltrials.gov # NCT05228782). The theoretical framework for the original HOW R U? intervention was based on “peer support”—the “provision of knowledge, experience, emotional or practical help by someone sharing common characteristics”—which facilitates the sharing of common experiences and empathetic communication [4, 5].

A review of interventions for SIL found that those based on a theoretical framework, such as the theory of generativity, are more likely to improve outcomes [7]. Further, Fakoya et al. highlighted that “there is no one-size-fits-all approach to loneliness interventions” [8]. Thus, there is a need to assess the effectiveness of different types of interventions to meet differing experiences of loneliness.

Compared to peer support interventions from similar-aged volunteers, “intergenerational” interventions pair members of younger and older generations with a focus on mutual benefit [9]. These intergenerational interventions are based on Erikson’s theory which posits that generativity, defined as “the interest in establishing and guiding the next generation”, is an important need that remains so into older adulthood (see Appendix 1 for further details of this theoretical framework) [10]. Evidence suggests this may produce reciprocal benefits for the client and volunteers including improved productivity, increased social interaction, enhanced self-perceptions of generativity, and feelings of self-worth among its participants [7, 8]. In addition, recent research suggests that generativity may in itself be a modifiable construct potentially yielding benefits in mental and physical well-being [10].

The current protocol is part of a program of research following the Obesity-Related Behavioral Interventional Trials (ORBIT) model whereby complex behavioral interventions are systematically refined to prevent premature evaluation, similar to the development of pharmaceuticals [11].

### Study purpose

Our primary outcomes are to assess whether the intergenerational HOW R U? intervention (1) reduces SIL more than the waitlist control group at study week 12 and (2) reduces SIL more than the current same-generation peer support HOW R U? intervention at study week 12.

Secondary outcomes include assessing:

1. The impact of the intergenerational HOW R U? intervention on (i) SIL at study week 24, (ii) depression, (iii) quality of life, (iv) functional status, (v) generativity, (vi) participant’s perceived self-benefit, and (vii) participant’s perceived benefit for the volunteer delivering the HOW R U? intervention at study weeks 12 and 24.
2. Participant stated preferences for intergenerational versus same-generation peer support HOW R U? intervention prior to randomization. Note, participants will be randomized regardless of their intervention preference.

## Methods/design

This protocol describing a three-arm RCT was developed following SPIRIT guidelines for reporting trial protocol and a SPIRIT guidelines checklist is attached [12]. Per SPIRIT guidelines, trial registration information is summarized in Tables 1 and 2.

**Table 1:** SPIRIT schematic diagram of enrolment, interventions, and assessments. Listed assessments are completed for all intervention groups

	**Enrolment**	**Allocation**	**Post-allocation**		
Timepoint	***Pre-intervention***	**Time 0**	***Intervention (12 weeks)***	***12 weeks’ follow up***	***24 weeks’ follow up***
Enrolment:					
Eligibility screen	X				
Informed consent	X				
Allocation		X			
Interventions:					
* Intergenerational intervention*			X		
* Peer-support intervention*			X		
* Waitlist control*					
Assessments:					
* Demographic data*	X			X	X
* De Jong Loneliness Scale*	X			X	X
* Lubben’s Social Network Scale*	X			X	X
* Geriatric Depression Scale*	X			X	X
* Brief Alzheimer’s Screening Test*	X			X	X
* EQ-5D-5L*	X			X	X
* Older Americans Resources Scale*	X			X	X
* Loyola Generativity Scale*	X			X	X
* Perceived benefit by the participant*	X			X	X
* Volunteer benefit perceived by participant*	X			X	X

**Table 2:** World Health Organization Trial Registration Data Set

Data Category	Information
Primary registry and trial identifying number	Clinicaltrials.gov NCT05998343
Date of registration in primary registry	24 July, 2023
Secondary identifying numbers	Protocol ID:21-0074E
Source(s) of monetary or material support	N/A
Primary sponsor	N/A
Secondary sponsor(s)	N/A
Contact for public queries	Jacques Lee, MD Jacques.lee@sinaihealth.ca
Contact for scientific queries	Jacques Lee, MD Jacques.lee@sinaihealth.ca
Public title	Intergenerational Program for Social Isolation and Loneliness in Older Adults Discharged From the Emergency Department
Scientific title	Impact of an intergenerational program to improve loneliness and social isolation in older adults initiated at the time of emergency department discharge: Study protocol for a three-arm randomized controlled trial
Countries of recruitment	Canada
Health condition(s) or problem(s) studied	Social isolation, loneliness
Intervention(s)	1. HOW R U? intervention delivered by same-generation peer volunteer 2. HOW R U? intervention delivered by intergenerational volunteer 3. No intervention
Key inclusion and exclusion criteria	Inclusion Criteria 1. Community-dwelling person 70 years of age and older receiving care from the ED, Family Medicine or Geriatric clinics at the two participating sites (MSH, NYGH) 2. Baseline de Jong loneliness score of 2.0 will be required for participating in the trial Exclusion Criteria 1. Age less than 70 years of age 2. Patients with communication problems (critically ill, unconscious, language barrier, speech impairment or otherwise unable to provide consent) or admission to a hospital for greater than 72 h) 3. Patients with severe cognitive impairment or those with living in nursing homes who are dependent on others for their activities of daily living 4. Patients without any mobile phone or landline Volunteer Inclusion Criteria 1. Peer-support volunteers will be 60 years of age or older 2. Intergenerational volunteers will be between 19–39 years of age
Study type	Interventional Allocation: Randomized interventional model. Parallel assignment. Outcome assessor masking Primary purpose: Supportive Care
Date of first enrolment	November 1, 2021
Target sample size	141
Recruitment status	Recruiting
Primary outcome(s)	1. Change in loneliness using De Jong Gierveld 6-item Loneliness Scale from baseline to 12 weeks
Key secondary outcomes	1. Change in loneliness using De Jong Gierveld 6-item Loneliness Scale from baseline to 24 weeks 2. Change in perceived social support using Lubben Social Network Scale from baseline to 12 and 24 weeks 3. Change in mood using Geriatric Depression Scale from baseline to 12 and 24 weeks 4. Change in baseline cognition using “Brief Alzheimer’s Screening Test” from baseline to 12 and 24 weeks 5. Change in Quality of Life using Euro-Qual 5 Dimensions 5 Levels from baseline to 12 and 24 weeks 6. Change in Functional Status using Older Americans Resource Scale from baseline to 12 and 24 weeks 7. Change in self-perceptions of generativity using Loyola Generativity Scale from baseline to 12 and 24 weeks 8. Perceived benefit to participant at 12 and 24 weeks 9. Perceived benefit to volunteer at 12 and 24 weeks

### Study design and setting

We will conduct a prospective multi-centered three-arm, single-blind, superiority RCT. The study will recruit participants from three settings: (1) the ED of the Schwartz/Reisman Emergency Medicine Center, (2) the Granovsky-Gluskin Family Medical Centre at Mount Sinai Hospital, and (3) the ED of the North York General Hospital. All three sites are in Toronto, Ontario, Canada. Mount Sinai Hospital is an urban tertiary care center with an annual census of 65,000 ED patients. North York General is a university-affiliated academic community hospital with an annual census of 118,000 ED patients. For both hospitals, 17–20% of ED patients were 70 years and older over the last 3 years.

### Hypotheses

Our primary hypothesis is that participants who receive the intergenerational HOW R U? intervention will have superior outcomes compared to the waitlist control group and to the same-generation peer support HOW R U? intervention. Our second hypothesis is that participants with higher perceptions of generativity will have greater reductions in SIL. Third, we hypothesize that participants in the intergenerational intervention will have higher perceptions of generativity after 12 weeks than the same-generation peer support and waitlist control groups.

### Recruitment and training

Participants will be recruited from the EDs of Mount Sinai Hospital and North York General Hospital as well as the Granovsky-Gluskin Family Medical Centre at Mount Sinai Hospital. Same-generation peer support volunteers will be vetted and approved by the existing hospital volunteer programs at Mount Sinai Hospital and North York General Hospitals. We will also use a social media recruitment campaign if required. Volunteers for the intergenerational intervention group will be recruited via university student groups associated with the University of Toronto and Western University. Volunteers of both the same-generation peer support and intergenerational HOW R U? interventions will attend a standardized, interactive 3-h training session. Content discussed will include ageism, mental health and aging, empathetic listening, validation, available community supports, confidentiality, and safety protocols. HOW R U? uses a strength-based approach focusing on empowerment and the participant’s strengths as previously developed by Lowthian et al. [4].

### Inclusion and exclusion criteria

Participant inclusion criteria are as follows: (1) age 70 years and older and (2) a minimum baseline six-item De Jong loneliness score of 2/6, corresponding to loneliness or high risk of loneliness [13]. We will accept referrals from participating ED and clinics as well as self-referrals from participants to improve recruitment.

We will exclude individuals (1) living in nursing homes; (2) with significant cognitive impairment as assessed by the Adamis capacity assessment [14, 15]; (3) with difficulty communicating (i.e., critically ill, unconscious, unable to communicate in English, speech impairment or otherwise unable to provide consent); (4) without access to a telephone; and (5) admitted to hospital for > 72 h. Volunteers will be excluded if they are less than 19 years of age and, in the case of intergenerational volunteers, if they are over 39 years of age. Same-generation peer support volunteers less than 60 years of age will be excluded.

### Randomization and blinding

We will use a computerized number generator to randomize to the three study arms in blocks of three, stratified by referral source. This will be an open-label trial. We will use an outcome assessment blinding process developed in our previous trial: outcomes assessors are blinded to the intervention group and record the primary outcomes at the start of follow-up calls to prevent accidental unblinding. Participants are also instructed not to reveal which intervention to any research staff, and any instances of unblinding will be documented by the outcome assessor. No formal auditing process is implemented for this trial.

### Intervention

Following randomization, research assistants will attempt to match volunteers and participants based on (1) any gender preferences and (2) shared interests or hobbies. This matching process will serve to promote adherence to intervention protocols as will the standardized 3-h volunteer training session which will teach strategies for volunteers to encourage participant engagement. Finally, we have attempted to minimize the research burden on participants to further limit potential contributors to nonadherence. For example, there is no interim data collection between enrollment and completion of the study. We are monitoring adherence by logging the time and duration of phone calls between volunteers and participants. Note, as the study’s intervention is a social interaction and not a pharmaceutical trial, drug tablet returns and laboratory tests are not applicable.

#### Same-generation peer support intervention

The same-generation peer support intervention group will receive the current HOW R U? intervention delivered by similarly aged peer volunteers. The HOW R U? intervention consists of weekly 30-min telephone-support calls over 12 weeks. The focus of each interaction is a strength-based approach including encouraging the participant to identify goals to improve SIL such as integrating with community activities [4]. Volunteers will reassess these goals each week and will maintain progress logs for each participant consisting of session dates, session duration, sessions missed, and any participant drop-outs.

#### Intergenerational intervention

The intergenerational intervention group will receive the HOW R U? intervention over the telephone as described above and delivered by trained intergenerational volunteers. Unique to the intergenerational intervention, informal service-learning goals will be assigned to the student volunteers and simultaneously made known to their participants. These include reflecting on the older adult’s experience of navigating the healthcare system, identifying negative ageist perceptions and stereotypes associated with older adults and the aging process, and risk factors for SIL among older adults. The purpose of these additional objectives is to facilitate a sense of mutual benefit and self-perceptions of generativity in the older adult participants consistent with Erikson’s theory [16].

#### Waitlist control

The current standard of care is not to offer interventions for SIL. Thus, participants randomized to the control group will be assigned to a 24-week waitlist and not receive any interventions. Following the assessment of primary and secondary outcomes after a 24-week period, control group participants will exit the current trial but will be offered their choice of the intergenerational or same-generation peer support HOW R U? intervention. There are no other criteria specified in this trial for concomitant care that is permitted/prohibited.

#### Criteria for discontinuing or modifying interventions

As this is a minimal-risk study, no modification or discontinuation of interventions is anticipated. Participants voluntarily participate in the intervention and can withdraw their consent and exit the study at any time. There are currently no established criteria for modifying allocated interventions for a given trial participant.

### Data collection

Research assistants will collect data for all groups over the telephone. Initial screening of potential participants by research staff involves the six-item De Jong loneliness scale which is described in further detail below. We will then collect demographic data including age, gender, living arrangement, pet ownership, educational attainment, technology profile index (modified), and baseline assessments of primary and secondary outcomes (see the “Outcome measures” section) from eligible participants who provide informed consent. Trained research staff will obtain verbal informed consent for this study as approved by all participating institutional REBs (see Appendix 2 for the informed consent model). Research staff are not involved in the clinical management of participants to avoid the perception of coercion. In order to allocate patients to specific arms, a statistician independent of the trial management will use a computer procedure to generate a variable block randomization list with blocks varying between four and eight. Note, as in our previous trial, research staff involved in outcome assessment will be blinded to randomization status. After completion of the 12-week intervention, research assistants will reassess outcomes within 2 weeks (study week 12–14) and 12 weeks (study week 24). To minimize the potential Hawthorne Effect, study endpoints will be collected only upon completion of the intervention [17]. Personal health information is removed from all study databases. Deidentified data will be stored in a password-protected computer accessible only by the study’s research assistants and corresponding author and will remain confidential. As this is a minimal-risk study, no formal data monitoring committee was required by our REB. A study committee consisting of the principal investigator, coauthors, and statistician will have regular meetings once a quarter to discuss ongoing data collection, quality, and outcomes. Note, no interim analyses are planned in this minimal-risk study.

### Outcome measures

The primary outcome is loneliness as measured using the six-item De Jong loneliness scale after completion of the intervention (weeks 12–14 of the study). The De Jong scale has been extensively validated in older populations and is divided into four clinically relevant categories: 6—severe loneliness, 4–5—moderate-to-severe loneliness, 2–3—low loneliness, and 0–1—no loneliness [12].

The secondary outcomes are the following: (1) the de Jong loneliness Scale at 12 weeks post-completion of the intervention to assess sustainability (study week 24); (2) six-item Lubben’s Social Network Scale [18]; (3) five-item Geriatric Depression Scale to assess mood [19]; (4) Brief Alzheimer’s Screening Test to assess baseline cognitive performance [20]; (5) EQ-5D-5L to assess perceived health-related quality of life in the areas of self-care, mobility, usual activities, pain/discomfort, and anxiety/depression [21]; (6) the Older Americans Resource Scale to assess functional status [22]; (7) the Loyola Generativity Scale to assess generativity [23]; (8) perceived benefit by the participant; and (9) benefit to the volunteer as perceived by the participant. The standardized measurement instruments selected for the trial have been extensively validated in older adults. The Loyola Generativity Scale has demonstrated good interrater reliability as well as responsiveness in trials of older adults [24, 25]. To assess the secondary outcome of participant preference for same-generation peer support versus intergenerational HOW R U? intervention, we will use a 5-point Likert scale with the following options: “I strongly prefer peer support,” “I prefer peer support,” “neutral,” “I prefer intergenerational,” and “I strongly prefer intergenerational”. Please see Appendix 3 for full details.

### Sample size and power

Each group will consist of a sample of 47 subjects which will detect a 13.9–28.1% difference between the treatment and control groups in the proportion of people who improve by one category (e.g., from severe loneliness to moderate-to-severe loneliness) with a power greater than 80%. This assumes that up to 15% of control group participants may have a spontaneous improvement in loneliness categories. With these sample sizes, three comparisons may be made: intergenerational intervention to the control group, same-generation peer support intervention to the control group, and intergenerational to the same-generation peer support intervention.

### Analysis

All analyses will use SAS Version 9.4 (SAS Institute, Cary, NC, USA). A two-sample two-sided test of proportions will be used to assess the primary outcome of loneliness in the control group versus the intergenerational HOW R U?, and the control group versus the same-generation peer support HOW R U? intervention. Improvements in the binary outcome of the loneliness category of the De Jong Loneliness Scale will be assessed using a logistic regression model, and results will be reported as odds ratios with the corresponding 95% confidence intervals. The variance inflation factor (VIF) will be calculated to assess for multicollinearity before the logistic regression model is built such that if two variables are highly correlated (i.e., VIF > 4 or tolerance < 0.25), only one of the two variables is retained for the final model. To compare the secondary outcomes, they will be reported as means with corresponding 95% confidence intervals.

Potential missing data will be managed as follows. For logistic regression, participants with more than 15% missing data for confounding variables will be excluded from the analysis. Note that in the main trial, less than 5% of participants would have been excluded. For participants with less than 15% missing data, we will use imputation for confounding variables. Participants with missing outcome data will be excluded from analysis (i.e., lost to follow-up). Note that after two-thirds of the participants have been enrolled, we will assess the loss-to-follow-up rate and adjust the enrollment accordingly. For example, if the lost-to-follow-up rate is 20%, we will increase the enrolment by 20%. To manage protocol nonadherence, participants will be analyzed using intention-to-treat analysis regardless of their adherence to the protocol.

## Discussion

We describe a protocol for a three-arm RCT assessing the effectiveness of an intergenerational telephone support intervention compared to the same-generation peer support intervention and to a common control group in reducing SIL for the older adult population after discharge from the ED.

The ED represents an important but unexploited setting to screen for SIL and initiate social interventions. Prior studies have supported the benefit of intergenerational interventions in reducing SIL in older adults in long-term care homes as well as those who were solicited in the community, though these benefits have not been assessed in the post-ED discharge setting, nor have they been compared to the same-generation peer support interventions [7].

The aging process is experienced diversely by different individuals. Thus, a suite of interventions to address SIL may be beneficial. Intergenerational interventions are a potential alternative to peer support interventions for reducing SIL while potentially benefiting student participants as well [7, 26]. In addition, younger intergenerational volunteers could expand the pool to recruit volunteers and broaden the impact of any intervention. Assessing the effectiveness of the intergenerational HOW R U? intervention will inform whether it is beneficial in reducing SIL and whether it can be applied in the ED setting, and increase our understanding of generativity as a modifiable construct for the development of future social interventions.

Limitations of this protocol include the fact that it is restricted to being delivered in the English language and that it requires a telephone at minimum to participate, although greater than 90% of Canadians have access to a telephone including those among the lowest income quartile [27]. In addition, the defined age range of intergenerational volunteers (i.e., 19–39 years of age) is admittedly arbitrary, and intergenerational programs’ age ranges have historically been loosely defined in the literature [9, 28]. As such, the findings of this study are not generalizable to intergenerational programming involving volunteers of adjacent generations (i.e., age 39–59). Potential confounders of the primary outcome measures include the individual interpersonal relationships between participant and volunteer, as well as potential differences in preference and ultimate assignment to intergenerational or same-generation peer support intervention. A follow-up RCT could compare the impact of preference accommodation versus a random selection of social intervention on reducing SIL. In addition, while social desirability bias is known to complicate research on stigmatized conditions such as isolation and loneliness, the De Jong scale is an indirect measure of loneliness to minimize this bias. Furthermore, outcomes among the intervention and control subjects will be evaluated using the same scale following randomization, thus mitigating the impact of this bias.

Given the lack of studies on modifying generativity, one area of uncertainty is in the ability of the present intergenerational intervention design to increase self-perceptions of generativity. Few studies have looked at self-perceptions of generativity as a modifiable construct, and there is no standard method of promoting these self-perceptions used in the literature. However, the secondary outcome measure of “perceived benefit to the volunteer” may serve as an adjunct for measuring generativity [7]. Lastly, though not formally assessed in this RCT, further studies may measure the potential benefits of modifying attitudes towards aging in young volunteers participating in intergenerational interventions.

## Conclusions

This protocol is part of a program of research following the ORBIT model for complex behavioral interventions. As such, the findings of this three-arm RCT have been informed by our previous research and will inform future research exploring which intervention characteristics are most effective in reducing SIL for specific older adult subgroups. Future RCTs will assess whether the element of patient choice between different demographics of groups yields differences in reducing loneliness.

## Trial status

Protocol version: 2023-Oct-03 Version 2.

Date of Recruitment Start: November 1, 2021.

Approximate Date of Recruitment Completion: February 1, 2024.

## Figures and Tables

**Fig. 1:**
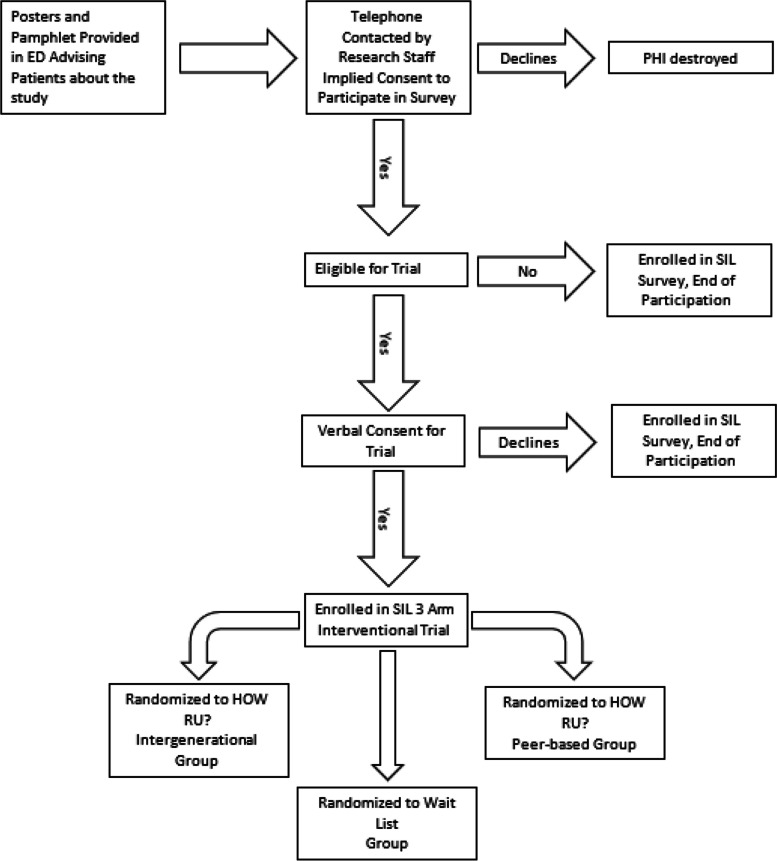
Proposed informed consent model for emergency department patients

## Data Availability

Not applicable.

## Appendix 1

### Theoretical framework of intergenerational intervention

The modified intergenerational HOW R U? intervention is grounded in Erikson’s theory of psychosocial development which posits that individuals progress through psychological stages each with a developmental crisis that is either successfully resolved or contributes to negative personality development. During middle adulthood, generativity, defined by Erikson as “the interest in establishing and guiding the next generation,” or in other words “the need to be needed” is the primary goal to be met at this stage [29]. This generativity manifests as mentoring, caretaking, parenting, and ultimately contributing meaningfully to the lives of others. Failure to do so leads to “stagnation.” Eriksonian theory also suggests that providing a renewed sense of purpose may result in “engaging in self-management to remain autonomous rather than passively allowing others to manage their care” [16].

Erikson further believed that the need for generativity extends well into late life as “grand generativity,” defined as a continuation of the need to assist the next generation into older adulthood to provide role modeling to young people while simultaneously benefiting from interactions with the young [30, 31]. This type of reciprocal relationship is encapsulated in intergenerational programs, defined as involving social interactions among members of the younger and older generations and intergenerational exchange [32]. Whereas volunteering places emphasis on the unidirectional benefit to the recipient, intergenerational service learning promotes equal emphasis of benefit to the volunteer and participant and, in doing so, enhances participants’ self-perceptions of generativity. In turn, greater perceptions of generativity are associated with improved mental and physical well-being and lower levels of negative affect and depressed mood [7]. Thus, in keeping with Eriksonian theory, adding service-learning goals and making them known to both student volunteer and participant promote self-perceptions of generativity among participants that are hypothesized to reduce feelings of SIL.

## Appendix 3

### Description of assessment tools used for outcome measures

#### Primary outcome measure scales

1. De Jong 6-item Loneliness Scale: The De Jong scale has been extensively validated in older populations and is divided into four clinically relevant categories: 6—severe loneliness, 4–5—moderate-to-severe loneliness, 2–3—low loneliness, and 0–1—no loneliness [12].

#### Secondary outcome measure scales

1. Lubben’s 6-item Social Network Scale: The Lubben’s Social Network Scale is a measure of perceived social support received from family and friends and consists of six items each of which is scored from 0 to 5: none = 0, one = 1, two = 2, three or four = 3, five through eight = 4, nine or more = 5. The total score ranges from 0 to 30, and a score of 12 or lower indicates a status of “at-risk” for social isolation [33]. The Lubben’s Social Networks Scale has been shown to be a reliable and valid instrument among older adults [33].
2. 5-item Geriatric Depression Scale: The 5-item Geriatric Depression Scale is a scale of mood that is extensively validated in older populations to screen for depression [19]. Scores range from 0 to 5. A score of 0–1 is considered normal suggesting that the patient is not depressed, and a score of 2 or higher is considered indicative of possible depression with higher scores indicating higher severity of depression [19].
3. Brief Alzheimer’s Screening Test: The Brief Alzheimer’s Screening test is a test that has been validated retrospectively on a cohort of older adults and used to assess cognitive performance and to screen for dementia [34]. There are four questions which are scored correct or incorrect and inputted numerically into an algebraic equation to distinguish normal older adults with those with dementia. A score of 26 or less is consistent with dementia [34].
4. Euro-Qual 5 Dimensions 5 Levels (EQ-5D-5L): The EQ-5D-5L is used to assess perceived health-related quality of life in the areas of self-care, mobility, usual activities, pain/discomfort, and anxiety/depression [21]. In each dimension, quality of life can be measured from 1 (best quality of life) to 5 (worst quality of life), giving a possible score of 5–25. A higher score indicates a worse quality of life. This instrument has been used and shown to be feasible in the elderly population [35].
5. Older Americans Resource Scale (OARS): The Older Americans Resource Scale is used to assess functional status and has been shown validity and reliability in the elderly population [36]. It consists of 7 activities of daily living (ADL) questions and 7 instrumental activities of daily living (IADL) all rated as “without any help” (2 points), “with some help” (1 point), or “completely unable” (0 points). The ADL and IADL scores can be used separately or combined to produce an overall OARS score from 0 to 28.
6. Loyola Generativity Scale (LGS): The Loyola Generativity Scale measuring generativity is a 20-item scale with each question answered 0 = statement never applies to you, 1 = statement only occasionally or seldom applies to you, 2 = statement applies to you fairly often, or 3 = statement applies to you very often or nearly always. The higher the score, the greater the sense of generativity. The LGS has demonstrated good interrater reliability as well as responsiveness in trials of older adults [24, 25].

## Abbreviations

ED: Emergency department
HOW R U?: HOspitals and patients WoRking in Unity
ORBIT: Obesity-Related Behavioral Intervention Trials (ORBIT)
RCT: Randomized controlled trial
REB: Research Ethics Board
ADLs: Activities of daily living

## Declarations

### Dissemination policy

The outcomes of this trial will be disseminated in ClinicalTrials.gov and in a peer-reviewed journal. Full protocols, statistical code, and datasets collected in the study are not publicly available. The results of the study can also be shared with any interested study participants on reasonable request to the principal investigator.
